# Plasma Fatty Acids and Quantitative Ultrasound, DXA and pQCT Derived Parameters in Postmenopausal Spanish Women

**DOI:** 10.3390/nu13051454

**Published:** 2021-04-25

**Authors:** Raúl Roncero-Martín, Ignacio Aliaga, Jose M. Moran, Luis M. Puerto-Parejo, Purificación Rey-Sánchez, María de la Luz Canal-Macías, Antonio Sánchez-Fernández, Juan D. Pedrera-Zamorano, Fidel López-Espuela, Vicente Vera, Purificación Cerrato-Carretero, Jesús M. Lavado-García

**Affiliations:** 1 Metabolic Bone Diseases Research Group, Nursing and Occupational Therapy College, University of Extremadura, 10003 Caceres, Spain; rronmar@unex.es (R.R.-M.); lmpuerto@unex.es (L.M.P.-P.); prey@unex.es (P.R.-S.); luzcanal@unex.es (M.d.l.L.C.-M.); jpedrera@unex.es (J.D.P.-Z.); fidellopez@unex.es (F.L.-E.); puricc@yahoo.es (P.C.-C.); jmlavado@unex.es (J.M.L.-G.); 2 Department of Conservative Dentistry and Prostheses, Faculty of Dentistry, Complutense University, 28040 Madrid, Spain; i.aliaga@pdi.ucm.es (I.A.); vveragon@ucm.es (V.V.); 3 Servicio de Tocoginecología, Complejo Hospitalario de Cáceres, 10004 Caceres, Spain; gineantonio@gmail.com

**Keywords:** fatty acids, *n*-3 PUFAs, bone mineral density, osteoporosis, postmenopause

## Abstract

Different factors may contribute to the development of osteopenia or osteoporosis. Fatty acids are key nutrients for health, and a number of studies have reported an association between bone mineral density (BMD) and fatty acid intake. We aimed to investigate the relationships between serum levels of different fatty acids and bone parameters determined by quantitative bone ultrasound (QUS), peripheral quantitative computed tomography (pQCT), and dual-energy X-ray absorptiometry (DXA) in a sample of Spanish postmenopausal women. We enrolled a total of 301 postmenopausal women (median age 59 years; interquartile range (IQR) 7) in this study. All participants underwent full densitometric screening, including calcaneal quantitative ultrasound (QUS), peripheral quantitative computed tomography (pQCT), and dual-energy X-ray absorptiometry (DXA), as well as plasma fatty acid measurement. After adjustment for potential confounders, plasma *n*-3 polyunsaturated fatty acid (PUFA) levels correlated with BMD in the spine (r = 0.150; *p* = 0.014) and femoral neck (r = 0.143; *p* = 0.019). By multiple linear regression, an independent statistically significant positive relationship was observed between BMD in the spine and BMI (β = 0.288; *p* = 0.001) as well as total plasma *n*-3 PUFAs (β = 0.155; *p* = 0.009). The plasma *n*-3 PUFA level was also a significant and positive predictor of BMD at the femoral neck (β = 0.146; *p* = 0.009). Independent risk factors for low BMD (T-score ≤ 1) were determined by logistic regression analysis, and a relatively high level of plasma *n*-3 PUFAs (OR = 0.751; 95% CI 0.587-0.960, *p* = 0.022) was identified as a protective factor against low bone mass. In this single-center sample of Spanish postmenopausal women, we reported a significant positive and statistically independent association between BMD and plasma levels of *n*-3 PUFAs.

## 1. Introduction

Several diverse factors may contribute to the development of osteopenia/osteoporosis. Some of the most important are a sedentary lifestyle, inadequate nutrient consumption, inflammation, and genetic factors. The essential fatty acids are nutrients of key importance for health. Previous reports regarding the influence of diet on bone health in healthy populations established a link from fatty acid intake to total bone mineral density (BMD) through a number of mechanisms that promote bone formation [1,2,3].

The long-chain *n*-3 polyunsaturated fatty acids (*n*-3 PUFAs) α-linolenic acid (ALA) is mainly consumed from food sources, such as various nuts and seeds (walnuts, flaxseeds, chia seeds) and vegetable oils (linseed oil, canola oil, soybean oil), while eicosapentaenoic acid (EPA) and docosahexaenoic acid (DHA) are key fatty acids that are found almost exclusively in marine foods such as fatty fish [4]. *n*-6 PUFAs (linoleic acid (LA) and arachidonic acid (AA)) and *n*-3 fatty acids are of specific interest because they contribute to the structure and function of the phospholipid bilayers that constitute cell membranes and because they are precursors of eicosanoids, comprising prostaglandins, leukotrienes, and thromboxanes, with hormone-like activities [5]. *n*-3 PUFAs have the potential to benefit the bones, as increased consumption rates appear to be linked with increased BMD [6,7,8,9]. Mechanisms postulated for the effect of *n*-3 PUFAs on bone health include an indirect effect based on enhancing intestinal calcium uptake, together with direct effects on bone turnover based on affecting the activity of osteoblasts and osteoclasts [10,11,12,13]. It has been suggested that the intake of polyunsaturated lipids may have an influence on bone mineral accrual and BMD and may even play a relevant role in the prevention of fragility fractures [3,14,15,16]. The strongest evidence comes from observational studies that have reported how total PUFA intake, particularly *n*-3 and *n*-6 PUFA intake, may increase BMD and even reduce fracture risk [3,17,18,19,20]. Nevertheless, the literature is very limited, particularly with regard to monounsaturated fatty acids (MUFAs) and saturated fatty acids (SFAs) and their association with BMD or the risk of fracture [21]. A limited number of studies have addressed the role of these fatty acids in particular, finding that monounsaturated fatty acid intake [14] may decrease total fracture risk.

However, the relationship between dietary PUFA consumption and hip fracture risk has shown controversial results in several observational studies, some with large sample sizes. The Nurses’ Health Study (NHS) followed postmenopausal women for 24 years and showed a statistically significant decrease in age-adjusted hip fracture risk in women with higher total *n*-3 PUFA intake compared to those with lower intake [15]. In a further observational cohort of over 135,000 postmenopausal US women participating in the Women’s Health Initiative (WHI), total (*n*-3 and *n*-6) PUFA intake was also assessed and was found to be linked to a reduction in overall fracture risk after an average follow-up of 7.8 years, suggesting that an increased intake of certain *n*-3 PUFAs such as ALA and the intake of total *n*-6 PUFAs would be related to a reduced risk of fragility fractures [17]. However, as it has been indicated, these results contradict other available findings regarding the intake of fatty acids and their association with fractures and BMD. In most of the studies demonstrating an association, the authors note that it is not possible to determine the causality of these associations due to the observational nature of the studies [9,18], potentially making the observed results a consequence of residual confounding or reverse causality, in which case the inferred causality would be spurious.

Serum fatty acids circulating levels are ultimately determined by dietary consumption and biosynthesis. For *n*-3 and *n*-6 PUFAs, the percentages of LA, AA, EPA and DHA in total plasma fatty acids, are acceptable marker of their habitual consumption, but the percentage of ALA is not [22,23,24]. Long-chain *n*-6 and *n*-3 PUFA plasma concentrations are not affected by the intake levels of their precursors, LA and ALA [25]. This may explain some of the controversial findings regarding the intake of these fatty acids and their relationship to bone health. In this study, we assessed the relationships between serum levels of different PUFAs (*n*-6 and *n*-3), MUFAs and SFAs with bone density determined by quantitative bone ultrasound (QUS), peripheral quantitative computed tomography (pQCT), and dual-energy X-ray absorptiometry (DXA) in a sample of postmenopausal Spanish women.

## 2. Materials and Methods

### 2.1. Subjects

Subjects: In this cross-sectional study, a total of 301 postmenopausal women (median age 59 years; interquartile range (IQR) 7) that resided in the Urban Health District of Cáceres, Extremadura, Spain were enrolled from January 2019 to December 2019 in the local area from both primary and specialty care settings. Participants were recruited by convenience sampling from clinics. The participants underwent full densitometric screening, including calcaneal quantitative ultrasound (QUS), peripheral quantitative computed tomography (pQCT), and dual-energy X-ray absorptiometry (DXA). To be eligible for this study, these women were all required to be community residents of white European descent and not to have been diagnosed with functional mental or physical disabilities by either their current primary care physician or a specialist actively participating in their medical care. They were not prescribed any type of medication that might disrupt calcium metabolism (e.g., corticosteroids, oral anticoagulants, antipsychotics, etc.) and had no conditions, including those presumed to be potentially involved in disorders of mineral metabolism (diabetes mellitus, liver disease, renal osteodystrophy, or parathyroid, thyroid, adrenal, or ovarian disease), that would interfere with calcium metabolism. The subjects were all active, although none of them played any sport professionally The Ethical Advisory Committee of the University of Extremadura endorsed this study (protocol code 84/2018 and date of approval 6 July 2018). All the participants gave written informed consent in accordance with the 1975 Declaration of Helsinki.

### 2.2. Anthropometry

Height was measured using a Harpenden stadiometer with a mandibular plane that was parallel to the ground, while weight measurements were obtained with a biomedical precision scale. Height was accurately assessed to the nearest centimeter, and weight was measured to the nearest 100 g. All these measurements were performed while the subjects were only lightly clothed and shoeless. Alcohol consumption was reported to be occasional and did not exceed 100 mL/day. Body mass index (BMI) was computed as weight in kilograms divided by the square of height in meters (kg/m^2^).

### 2.3. Dietary Assessment

All women who participated in this study were provided with a 131-item comprehensive 7-day food frequency questionnaire. Food was quantified using a dietetic scale, measuring cups, and spoons. The questionnaire used was self-reported. The response rate was 91.3%. This questionnaire has been used extensively in the literature [26]. Nutrient and energy intake values were assessed according to the Spanish food composition database [27].

### 2.4. Quantitative Bone Ultrasound

Heel bone characteristics were evaluated with a GE Sahara (Hologic, Bedford, MA, USA) quantitative ultrasound (QUS) device. This apparatus acquires two key parameters: broadband ultrasound attenuation (BUA), expressed in dB/MHz, provides a measure of ultrasound decay with the incident frequency of the sound of the waves, and the speed of sound (SOS), expressed in meters per second, reflects the time necessary for the ultrasound waves to travel a given distance across the calcaneal bone.

### 2.5. Bone Densitometric Determinations

Peripheral quantitative computed tomography (pQCT) scans of the nondominant distal forearm were performed with a Stratec XCT-2000 scanner (Stratec Medizintechnik, Pforzheim, Germany). The equipment was positioned at the distal end of the nondominant forearm, and 4% of the forearm length was scanned. The XCT-2000 measurement data were processed with version 5.50 of the software supplied by the supplier. A pQCT scan provides a measure of volumetric bone mineral density and discriminates between trabecular bone and cortical bone.

Women in this study also underwent bone densitometry by dual-energy X-ray absorptiometry (DXA) of the lumbar spine (L2-L4, L2, L3, L4) and hip (left femoral neck and femoral trochanter) using a Norland XR-800 densitometer (Norland at Swissray, Fort Atkinson, WI, USA). All BMD measurements were given as the quantity of minerals divided by the scanned area (g/cm^2^). Participants were classified into the osteopenia, osteoporosis or normal group on the basis of the T-score at either the femoral neck or the spine (L2-L4).

The coefficient of variation (CV%) was below 2% in all bone measurements. We scanned an anthropomorphic pQCT phantom in each session to guarantee quality.

### 2.6. Determination of Plasma Fatty Acids

Fasting blood samples were collected, and plasma was stored at −80 °C until further analysis. Lipids were extracted from plasma samples, and fatty acids were isolated and separated by gas chromatography with flame ionization detection (GC-FID). GC–FID analysis was conducted using a Bruker Scion 456 GC equipped with a flame ionization detector (FID) and a DB-225 ms (Agilent Technologies) capillary column (30 m × 0.25 mm i.d., 0.25 µm film thickness), high-purity helium as the carrier gas, and a 0.2 µL injection volume, using a split/splitless capillary injection system at 220 °C with a split ratio of 100:1. The temperature program was as follows: initial temperature 140 °C for 1 min, increase by 10 °C/min to 200 °C, hold for 10 min, increase by 5 °C/min to 220 °C, and hold for 30 min. The total analysis run time was 51 min. A total of 17 different fatty acids were assessed: C8:0 (caprylic acid), C10:0 (decanoic acid), C12:0 (lauric acid), C14:0 (myristic acid), C16:0 (palmitic acid), C18:0 (stearic acid), C20:0 (arachidic acid), C22:0 (docosanoic acid), C24:0 (lignoceric acid), C16:1 (palmitoleic acid), C18:1 cis (n9) (oleic acid), C22:1 (n9) (erucic acid), C18:3 (n3) (α-linolenic acid, or ALA), C20:5 (n3) (eicosapentaenoic acid, or EPA), C22:6 (n3) (docosahexaenoic acid, or DHA), C18:2 (n6) (linoleic acid, or LA) and C20:4 (n6) (arachidonic acid, or AA). The average coefficient of variation was ∼60%.

### 2.7. Statistical Analysis

The results are reported as medians with interquartile ranges and frequency counts with percentages, unless otherwise specified. Continuous variables were analyzed using the Kruskal–Wallis test, the Mann–Whitney U test, and the Quade test, while categorical variables were analyzed using the chi-square test or Fisher’s exact test as appropriate. Pairwise comparisons between each independent group were analyzed by Dunn’s test with Bonferroni adjustment. The bivariate correlation analysis was carried out by calculating Spearman’s correlation coefficient and then adjusting (partial correlations) for potential confounding factors. Given that several variables included in this study did not fulfill the normality criteria (by the Kolmogorov–Smirnov test) for the multiple linear regression study, a two-step method was used to normalize the data prior to statistical analyses [28]. The variables included in the modeling were age (years), time since menopause (years), BMI (kg/m^2^), vitamin D (mcg/day), calcium intake (mg/day), energy (kcal/day), total plasma *n*-3 PUFAs, total plasma *n*-6 PUFAs, plasma *n*-6/*n*-3 ratio, total plasma SFAs, total plasma MUFAs, and total plasma PUFAs. Logistic regression was used to assess the probability of patients reaching an at-risk state for low bone mass, defined as a T-score < −1. The logistic regression included age (years), time since menopause (years), BMI (kg/m^2^), vitamin D (mcg/day), calcium intake (mg/day), and energy intake (kcal/day).

For all statistical analyses, a two-sided *p* value ≤ 0.05 was considered statistically significant. All analyses were performed with SPSS software (version 24.0, IBM Corp., Armonk, NY, USA).

## 3. Results

### 3.1. Descriptive Data

The anthropometric, biological and dietary characteristics studied in the group of women are shown in Table 1. Participants were grouped by diagnosis of osteopenia/osteoporosis based on WHO T-score criteria, and these groups were not significantly different in weight, age at menarche or years since menopause (*p* > 0.05). A significant difference was observed in BMI, which was significantly increased in the group of healthy women (*p* < 0.05). Overall, 80.7% (*n* = 243) of the participants were nonsmokers. No differences were observed in the intake of vitamin D, calcium, or energy according to the dietary questionnaire (*p* > 0.05).

The fatty acid profile of the study participants is shown in Table 2. A total of 17 different fatty acids were quantified. When subjects were categorized based on osteopenia/osteoporosis criteria, statistically significant differences were observed in the levels of C12:0 (lauric acid) (*p* = 0.029), C16:0 (palmitic acid) (*p* = 0.018), C24:0 (lignoceric acid) (*p* = 0.043), C18:1 cis (n9) (oleic acid) (*p* = 0.013), and total plasma MUFAs (*p* = 0.016). No statistically significant differences were observed in the levels of plasma total *n*-3 PUFAs, plasma total *n*-6 PUFAs, plasma *n*-6/*n*-3 ratio, plasma total SFAs, or total PUFAs (*p* > 0.05 in all cases). The statistically significant differences observed remained after adjusting for potential confounding factors in C16:0 palmitic acid (*p* = 0.036), C24:0 lignoceric acid (*p* = 0.042), and C18:1 cis (n9) oleic acid (*p* = 0.045).

### 3.2. Bone Parameters and Fatty Acid Plasma Levels

In order to explore the potential role of *n*-3 PUFAs in determining bone density, participants were classified based on the tertile of plasma *n*-3 PUFA levels (Table 3). This analysis revealed statistically significant differences in cortical volumetric density (*p* = 0.048) and BMD in the total spine (*p* = 0.011), L2 (*p* = 0.023), L3 (*p* = 0.033), and L4 (*p* = 0.004). The Z-score and T-score at the lumbar spine also showed statistically significant differences between the study groups (*p* < 0.05 in both cases). Regarding the hip, statistically significant differences were observed at the femoral neck in terms of the T-score (*p* = 0.042) as well as the Z-score (*p* = 0.035). We then proceeded to adjust this analysis for potential confounding factors present in the study sample, such as age; years since menopause; BMI; and intake of vitamin D, calcium, and energy. Differences observed in cortical volumetric BMD remained statistically significant (*p* = 0.013), as did those reported at the lumbar spine (*p* = 0.043) and L4 (*p* = 0.021). Previously reported differences in spine T-score and BMD at the femoral neck level were no longer statistically significant after adjustment (*p* > 0.05). Across all scores, the bone parameters determined to have statistically significant differences were highest in the tertile with the highest plasma level of *n*-3 PUFAs.

A similar analysis was performed on plasma *n*-6 PUFA levels. Participants were classified into tertiles according to their plasma *n*-6 PUFA concentration; the results are shown in Table 4. Statistically significant differences were observed in the SOS as determined by QUS (*p* = 0.045), with the highest tertile of plasma *n*-6 PUFAs being significantly higher than the middle tertile. This observed difference did not remain statistically significant after adjustment for potential confounding factors.

The ratio of *n*-6 to *n*-3 PUFAs was also studied, in addition to its association with the bone parameters analyzed (Table 5). After participants were classified according to the tertile of the *n*-6/*n*-3 PUFA ratio, statistically significant differences in lumbar Z-score were observed (*p* = 0.016), but these differences did not remain after adjustment for potential confounding factors (*p* = 0.166).

The role of the studied SFAs is shown in Table 6. After the participants were classified according to their plasma SFA tertiles, no statistically significant differences between groups were observed in any of the groups studied.

Table 7 shows the results for the studied MUFAs. After the participants were classified on the basis of tertiles, statistically significant differences were observed in total (*p* = 0.02), trabecular (*p* = 0.014) and cortical (*p* = 0.014) bone morphometry. These statistically significant differences indicated a smaller area (mm^2^) in those women belonging to the highest tertile of plasma MUFAs and remained statistically significant after adjustment for potential confounding factors (Table 7). In the group of women with high plasma MUFAs, the percentage of osteopenic women was also significantly increased.

When all measured PUFAs were pooled and participants were classified based on their plasma tertile (Table 8), statistically significant differences were observed in the SOS as determined by QUS (*p* = 0.004), but these differences did not remain statistically significant after adjustment for potential confounding factors.

### 3.3. Correlation Study

To further investigate the associations between BMD in the women studied and the fatty acids studied, we conducted an analysis using bivariate correlations and then partial correlations adjusted for age; years since menopause; BMI; and intake levels of vitamin D, calcium, and energy. The results are shown in Table 9. Statistically significant positive correlations were observed between BMD and plasma *n*-3 PUFA levels at the lumbar spine (r = 0.157; *p* = 0.006), L2 (r = 0.143; *p* = 0.013), L3 (r = 0.128; *p* = 0.026 and L4 (r = 0.178; *p* = 0.002) and femoral neck (r = 0.153; *p* = 0.008). Statistically significant and negative bivariate correlations were also reported between BMD and plasma *n*-6/*n*-3 PUFA levels at the lumbar spine (r = −0.121; *p* = 0.035), L4 (r = −0.156; *p* = 0.007) and femoral neck (r = −0.124; *p* = 0.031). Subsequent analysis of the partial correlations adjusted for the confounding factors studied showed that those observed for lumbar spine BMD and plasma levels of *n*-3 PUFAs remained statistically significant and positive (total spine (r = 0.150; *p* = 0.014), L2 (r = 0.141; *p* = 0.021), L3 (r = 0.129; *p* = 0.035), L4 (r = 0.170; *p* = 0.005) and femoral neck (r = 0.143; *p* = 0.019)). The bivariate correlations observed between plasma *n*-6/*n*-3 PUFA levels and the lumbar spine and femoral neck did not remain statistically significant (*p* = 0.086 and *p* = 0.073, respectively), although the correlation with L4 BMD did remain significant (r = −0.139; *p* = 0.023).

Partial correlations between the fatty acid profile and BMD at the spine or hip level are shown in Supplementary Table S1. After adjustment for potential confounders, the correlations observed between ALA and BMD L4 (r = 0. 123; *p* = 0.044) and between DHA and BMD at the lumbar spine (r = 0.135; *p* = 0.026), L2 (r = 0.148; *p* = 0.015), L3 (r = 0.122; *p* = 0.045), L4 (r = 0.136; *p* = 0.0.26), and femoral neck (r = 0.132; *p* = 0.031) remained statistically significant. A statistically significant partial correlation was also observed between erucic acid and BMD at the femoral neck level (r = −0.122; *p* = 0.035).

### 3.4. Multiple Linear Regression Study: Identification of Predictors

Independent relationships between BMD and the fatty acids studied were also explored using multiple linear regression including age (years), BMI (kg/m^2^), vitamin D (mcg/day), calcium intake (mg/day), energy (kcal/day), plasma total *n*-3 PUFA, plasma total *n*-6 PUFA, plasma *n*-6/*n*-3 ratio, plasma total SFA, plasma total MUFA and plasma total PUFA as explanatory variables. An independent statistically significant positive relationship was observed between BMD at the spine and BMI (β = 0.288; *p* = 0.001) as well as plasma total *n*-3 PUFAs (β = 0.155; *p* = 0.009), while a negative association was observed with age (β = −0.163; *p* = 0.006). The proposed model accounted for up to 11% of the variability associated with spine BMD in the study group (Table 10). Subsequently, we proceeded to repeat the analysis after classifying the participants based on the WHO osteopenia/osteoporosis criteria (Table 10). In the group of women with normal T-scores, only *n*-3 PUFA plasma levels (β = 0.273; *p* = 0.001) functioned as a positive predictor of BMD at the lumbar spine level, yielding a model that explained only 7% of the observed variability. In osteopenic women, the predictor included in the model was daily energy intake (β = 0.226; *p* = 0.011). Finally, in osteoporotic women, the model included a positive relationship with plasma *n*-3 PUFA levels (β = 0.290; *p* = 0.024) and a negative relationship with plasma MUFA levels (β = −0.411; *p* = 0.002). This model explained 28% of the variability observed in BMD at the lumbar level.

A parallel analysis was performed for BMD at the femoral neck level (Table 11). Overall, in the study sample, BMI β = 0.394; *p* ≤ 0.001) and plasma *n*-3 PUFA levels (β = 0.146; *p* = 0.009) were positive predictors of BMD at the femoral neck level. The global model accounted for a total of 18% of the reported variability (Table 11). After the subjects were categorized according to the WHO osteopenia/osteoporosis criteria, no predictors of BMD at the femoral neck level were identified in the group of osteoporotic women (Table 11). In the group of women with normal T-scores, the positive predictors observed were BMI (β = 0.320; *p* = 0.002) and plasma *n*-3 PUFAs (β =0.245; *p* = 0.015). This generated a model explaining 18% of the observed variability. In the group of osteoporotic women, only BMI β = 0.299; *p* = 0.001) was incorporated into the model as a positive predictor of BMD at the femoral neck level. The model was able to explain only 9% of the observed variability.

### 3.5. Logistic Regression Analysis for Low Bone Mass (T-score ≤ 1)

Independent risk factors for low BMD (T-score ≤ 1) were determined by logistic regression analysis. The analysis identified a higher BMI (OR = 0.893; 95% CI 0.841–0.948), *p* < 0.001) and a higher level of plasma *n*-3 PUFAs (OR = 0.751; 95% CI 0.587–0.960, *p* = 0.022) as protective factors against low bone mass. No other statistically significant factors were observed in the study sample (Table 12). Regarding the fatty acid profile and the risk of low bone mass, the results are shown in Supplementary Table S2, and no statistically significant results were observed.

## 4. Discussion

To the best of our knowledge, this is the first investigation of the association between the plasma fatty acid profile and bone density as determined by QUS, pQCT, and DXA. No consistent associations have been established between the levels of most of the fatty acids studied by QUS and pQCT. However, we have identified a stable association between the highest level of plasma *n*-3 PUFAs (ALA + EPA + DHA) and BMD at the level of the lumbar spine, but the individual role that the plasma level of each of these fatty acids might play in relation to BMD is difficult to interpret since each of these omega-3 fatty acids has different functions. Combined analysis reported here lead us to believe that they confirm previous findings obtained from observational studies in which it was observed that dietary intake of *n*-3 PUFAs acids was associated with better bone health and even with a reduction in the risk of fractures. Furthermore, these results lead us to consider that most of the fatty acids studied do not seem to have a notable impact and that future research should focus on deepening the role of *n*-3 PUFAs. Our results indicate that there is a consistent association with BMD at the lumbar level and probably a weaker association with BMD at the femoral neck area, but nevertheless, their higher concentration is associated with a lower risk of developing low bone mass in our sample of postmenopausal Spanish women.

Very few studies have established a relationship between the plasma fatty acid profile and bone density; most of these studies are focused on the study of *n*-3 and *n*-6 PUFAs and BMD determined by DXA. However, more data are available on associations between dietary intake of these fatty acids and BMD as if diets are rich in ALA + EPA + DHA then it is expected a higher level of plasma *n*-3 PUFAs and particularly, it has been shown that fatty acids are incorporated into plasma phospholipids after intake [29]. Hence, appropriate nutritional management, such as intake of *n*-3 fatty acids, may be a strategy to maximise peak bone mass in the female hip [30]. The interrelations of total and individual dietary PUFA intake with bone health are intricate [5,31,32,33] and may be further affected by nutrient-nutrient interactions, as well as by the ratio of *n*-6 to *n*-3 fatty acid intake. Given that the major dietary sources of *n*-3 PUFAs are foods of marine origin, different studies have examined the associations between their consumption and BMD. In 2011, Farina et al. [19] observed that the protective effects on BMD at the femoral neck level associated with a high intake of AA could depend on the intake levels of EPA + DHA, concluding that fish consumption can protect against bone loss and that these protective effects could also depend on a fine relationship between the levels of different fatty acids. The findings regarding fish consumption and bone health were further supported by QUS in premenopausal Spanish women [34]. In a further continuation of the original work by Farina et al. in 2012 [5], these authors analyzed relationships between plasma levels of certain fatty acids and BMD as well as risk of fracture. That study is believed to be the first work to address the association between plasma fatty acids and BMD determined by DXA. Their results suggested protective effects of plasma AA on femoral neck BMD in men, as well as protective effects of plasma AA against hip fracture risk in both women and men. Additionally, their results showed possible detrimental effects of plasma LA on BMD or hip fracture risk while identifying BMI as a possible confounding factor to be taken into account in these analyses. Their study suggested a possible more subtle effect of plasma DHA on BMD in older women and men. Overall, these results initially reported by the Farina et al. group have been confirmed by ours, suggesting that plasma levels of *n*-3 PUFAs appear to be associated with increased BMD at both the lumbar and hip levels, and DHA appears to correlate positively with BMD after adjustment for BMI and other potential confounders in our sample of postmenopausal women. Additional studies focusing on the analysis of osteoporotic fracture risk have also confirmed that higher plasma PUFA concentrations in old age are associated with a lower risk of osteoporotic fracture in men, but these findings were not replicated in women, as they did not reach statistical significance [35]. In men, plasma *n*-3 fatty acids and specifically plasma EPA seem to be relevant among PUFAs, while *n*-6 fatty acids in women may be associated with an increased risk of fracture.

However, focusing again on the nutritional aspect, it is true that not all studies have observed positive associations between PUFA intake and bone health. A long-term follow-up (two years) of patients with osteoarthritis of the knee supplemented with omega-3 fish oil did not demonstrate any efficacy of omega-3 fatty acids on bone loss in n = 202 Australian men and women aged ≥ 40 [36]. The findings in postmenopausal women enrolled in the Women’s Health Initiative are also remarkable [17]. Focusing on fracture risk, that study found that a higher dietary intake of SFAs was associated with a higher risk of hip fracture in postmenopausal women, while a higher intake of PUFAs and MUFAs was associated with a lower fracture risk. The authors reported no associations between total *n*-3 PUFA or ALA intake and fracture risk, but, strikingly, there was a slight increase in fracture risk associated with higher EPA + DHA intake. Regarding*n*-6 PUFAs, postmenopausal women with higher *n*-6 PUFA intake had a lower fracture risk. Although we did not determine the risk of fracture in our study, our results do seem to indicate that no negative effect of SFAs can be assumed, having detected only a small negative association between a higher plasma MUFA level and bone morphometry. These results, due to their preliminary nature, require additional studies to confirm that the association is not spurious. Neither have we observed negative or positive effects associated with a higher plasma level of *n*-6 PUFAs. Even the *n*-6/*n*-3 ratio, which is presumed to be more likely than individual classes of PUFAs to affect skeletal health [1,37,38], was not observed to have an effect in this study, although the Women’s Health Initiative observed that a higher ratio (>6.43) (the ratio in our sample was 8.28 (5.85)) offered discrete protection against fractures.

Subsequent results obtained by measuring *n*-3 fatty acids in red blood cells in a sample from the Women’s Health Initiative [39] suggested that more *n*-3 fatty acids in red blood cells (particularly ALA or EPA but not DHA) might be predictive of a lower risk of hip fracture. However, in this study, associations were not observed at baseline or after a 6-year follow-up. There was no relationship between red blood cell *n*-3 or *n*-6 fatty acids and BMD. The authors adjusted their analyses for potential confounders (alcohol consumption, total energy intake, total calcium intake, total vitamin D intake, and multivitamin use) and no changes were observed at longitudinal follow-up that would suggest an association between hip BMD and total *n*-3 or *n*-6 fatty acids in red blood cells. Similar results were observed when exploring the role of the *n*-6/*n*-3 ratio and the percentage change in BMD at longitudinal follow-up. By measuring *n*-3 FAs in red blood cells in Korean postmenopausal women [40], EPA, DHA, and total *n*-3 FAs were found to correlate with increased BMD of the femoral neck after adjusting for relevant confounders (age, years after menopause and height), whereas a higher *n*-6/*n*-3 FA ratio was correlated with decreased femoral neck BMD. Among young men, using an experimental approach similar to ours [3], it was also observed that concentrations of *n*-3 fatty acids, especially DHA, were positively associated with peak BMD in the whole body and spine and with bone accrual in the lumbar spine.

Regarding plasma fatty acids other than *n*-3 or *n*-6 PUFAs, in this study, statistically significant differences were observed according to the diagnosis of osteopenia/osteoporosis in C16:0 (palmitic acid), C24:0 (lignoceric acid) and C18:1 cis (n9) (oleic acid) after adjustment for potential confounding factors. For the first two, the level was significantly lower in women with osteopenia, while C18:1 cis (n9) (oleic acid) was more abundant in these women than in women with normal bone health according to the WHO osteopenia/osteoporosis criteria. There are previous results from a nutritional perspective indicating that the role of fatty acids in women’s bone health could be limited to the situation of osteopenia, with their role becoming secondary once osteoporosis is established [20]. In any case, our results are contrary to those recently published in which palmitic acid levels were associated with increased odds of low BMD in Chinese adult women, but our findings will require in-depth analysis in future studies, given that other types of associations were not observed in our study and therefore could represent some type of spurious association [41].

Evidence from in vivo studies on the role of different dietary sources of *n*-3 PUFAs on skeletal development and bone quality has shown that dietary *n*-3 PUFAs contribute to improved mechanical and morphometric properties of bone, and bone quality [42] with possible protective mechanisms against bone loss during ageing, associated with inhibition of inflammation associated with bone resorption mediated by NF-κβ, p38MAPK or JNK leading to the regulation of pro-inflammatory cytokine release [43]. However, the current evidence from human studies is limited at best. Thus, *n*-3 PUFA fatty acids could maintain bone density in postmenopausal women, but the mechanism is not known. Different mechanisms have been proposed. Essential fatty acids, by regulating eicosanoids, leptin and IGF-1, are linked to the regulation of both growth and bone status [44,45]. Other possible underlying determinants have also been suggested as potential meditators of the relationship between plasma fatty acids and BMD. They involve the regulation of lipid mediators, inflammation, or oxidative stress [21,46,47,48], a critical determinant of the decrease in bone strength and mass [49]. As some fatty acids have been found to be related to the potential to modulate the levels of both inflammatory cytokines (such IL-6, IL-1beta, and TNF-alpha) and eicosanoids [50,51,52], it has been hypothesized that they might lower the generation of free radicals and oxidative stress and somehow diminish the age-related loss of bone mass [49]. Among the possible molecular mechanisms that could be regulated by certain fatty acids, one of the most active fields of research concerns mechanisms linked to the regulation of prostaglandin E2 biosynthesis [53,54], which modulates osteoclastogenesis to accelerate bone resorption through activation of the nuclear factor kappa-B pathway [55] and through regulation of the synthesis of osteoprotegerin [12,13,56].

A strength of our study is that it includes the measurement of plasma fatty acids, which provides an unbiased measure of exposure and is more accurate than the information obtained from dietary surveys. We recognize that the main limitations are the cross-sectional design of the study, which prevents us from establishing cause-effect relationships, and the use of convenience sampling, which could lead to biases derived from the composition of the sample and also prevents the generalization of our results. Furthermore, plasma fatty acids were measured at a single time point, whereas it would be more accurate to perform a follow-up over time to establish previous exposure; nevertheless, it appears that plasma *n*-3 PUFA levels remain fairly consistent over time [57]. Another important limitation is the lack of analysis of plasma levels of C22:5*n*-6 which is a clear indicator of omega 3 fatty acid deficiency [58]. Finally, as the entire population in this study was Caucasian, this demographic restriction may affect the generalizability of the results of our study.

## 5. Conclusions

In this single-center sample of postmenopausal Spanish women, we reported a significant positive and statistically independent association between BMD and plasma levels of *n*-3 PUFAs that highlights the physiological and biochemical relevance of plasma total omega 3 fatty acids. Longitudinal observational or randomized controlled studies are needed to further investigate any effect of *n*-3 PUFAs on bone health.

## Figures and Tables

**Table 1:** Anthropometric, biological, dietary and lifestyle characteristics in the study sample.

	Total Sample (*n* = 301)	Normal (*n* = 103)	Osteopenia (*n* = 145)	Osteoporosis (*n* = 53)	
	Median (IQR); *n* (%)	Median (IQR); *n* (%)	Median (IQR); *n* (%)	Median (IQR); *n* (%)	*p*-Value
Age, years	59 (7)	58 (7)	60 (7)	60 (6)	0.071
Menarche age, years	13 (2)	12 (3)	13 (1)	13 (1)	0.842
Years since menopause, years	9 (9)	9 (10)	8 (9)	10 (10)	0.277
Weight, kg	66.4 (15.6)	70.6 (15.2) (a,b)	65.5 (13.8) (c)	58.1 (13.6)	<0.001
Height, m	1.58 (0.07)	1.59 (0.07)	1.58 (0.07)	1.57 (0.07)	0.078
BMI (kg/m^2^)	26.6 (5.6)	27.7 (5.9) (a,b)	26.6 (5.3) (c)	24.7 (4)	<0.001
BMI Classification					
Underweight (<18.5)	*n* = 1 (0.3%)	*n* = 1 (1%)	*n* = 0 (0%)	*n* = 0 (0%)	0.518
Normal weigth (18.5–24.9)	*n* = 98 (32.6%)	*n* = 23 (22.3%)(a,b)	*n* = 46 (31.7%) (c)	*n* = 29 (54.7%)	0.003
Overweight (25.0–29.9)	*n* = 135 (44.9%)	*n* = 47 (45.6%) (a,b)	*n* = 73 (50.3%) (c)	*n* = 15 (28.3%)	0.02
Obesity class I (30.0–34.9)	*n* = 50 (16.6%)	*n* = 21 (20.4%)	*n* = 23 (15.9%)	*n* = 6 (11.3%)	0.359
Obesity class II (35.0–39.9)	*n* = 12 (4%)	*n* = 8 (7.8%) (a,b)	*n* = 2 (1.4%) (c)	*n* = 2 (3.8%)	0.02
Obesity class III (≥40)	*n* = 5 (1.7%)	*n* = 3 (2.9%)	*n* = 1 (0.7%)	*n* = 1 (1.9%)	0.327
Waist circumference, cm	87 (14)	91 (17) (a,b)	87 (13) (c)	82 (13)	<0.001
Hip, cm	104 (12)	107 (13) (a)	104 (11) (c)	100 (14)	<0.001
Gravidity	2 (1)	2 (1)	2 (1)	2 (1)	0.256
Parity	2 (1)	2 (0)	2 (1)	2 (1)	0.44
Smoker					
No	*n* = 243 (80.70%)	*n* = 86 (83.5%)	*n* = 116 (80%)	*n* = 41 (77.4%)	0.597
Yes	*n* = 58 (19.3%)	*n* = 17 (16.5%)	*n* = 29 (20%)	*n* = 12 (22.6%)	
Fish intake (servings/week)	4 (3)	3 (4)	4 (3)	4 (3)	0.783
Vitamin D (mcrg/day)	7.4 (8.23)	7.4 (8)	7.6 (7.83)	7.8 (12.26)	0.707
Calcium intake, mg/day	944 (663)	930 (516)	959 (652)	882 (821)	0.908
Energy, kcal/day	2099 (869)	2087 (883)	2048 (860)	2204 (874)	0.892

Between-group comparisons were performed using the Kruskal Wallis test or the Fisher exact test as appropriate. (a) Poshoc analysis by Dunn’s test, *p* < 0.05 vs. osteoporosis group. (b) Poshoc analysis by Dunn’s test, *p* < 0.05 vs. osteopenia group. (c) Poshoc analysis by Dunn’s test, *p* < 0.05 vs. osteoporosis group.

**Table 2:** Plasma fatty acids profiles (%) in the studied sample.

	Total Sample (*n* = 330)	Normal (*n* = 103)	Osteopenia (*n* = 145)	Osteoporosis (*n* = 53)		
	Median (IQR)	Median (IQR)	Median (IQR)	Median (IQR)	*p* Value	Adjusted *p* Value *
C8:0 Caprylic acid	0.06 (0.09)	0.06 (0.07)	0.05 (0.09)	0.08 (0.07)	0.057	
C10:0 Decanoic acid	0 (0)	0 (0)	0 (0)	0 (0)	N/A	
C12:0 Lauric acid	0.07 (0.48)	0.06 (0.25) (a)	0.09 (0.55)	0.07 (0.08)	0.029	0.079
C14:0 Myristic acid	0.78 (0.28)	0.77 (0.26)	0.78 (0.31)	0.77 (0.25)	0.953	
C16:0 Palmitic acid	39.88 (3.69)	40.2 (3.36) (a)	39.28 (3.49) (b)	40.22 (4.39)	0.018	0.036
C18:0 Stearic acid	24.99 (5.04)	25.23 (3.86)	24.72 (5.93)	25.78 (3.53)	0.195	
C20:0 Arachidic acid	0.28 (1.08)	0.28 (0.89)	0.27 (1.11)	0.87 (1.21)	0.219	
C22:0 Docosanoic acid	0.34 (0.17)	0.33 (0.16)	0.35 (0.18)	0.33 (0.14)	0.796	
C24:0 Lignoceric acid	0.25 (0.15)	0.25 (0.14)	0.26 (0.17) (b)	0.22 (0.1)	0.043	0.042
C16:1 Palmitoleic acid	0.61 (0.39)	0.6 (0.39)	0.66 (0.4)	0.66 (0.38)	0.493	
C18:1 cis (n9) Oleic acid	10.16 (4.16)	9.75 (3.01) (a)	10.82 (4.47)	9.57 (3.85)	0.013	0.045
C22:1 (n9) Erucic acid	0 (0)	0 (0)	0 (0)	0 (0)	N/A	
C18:3 (n3) Linolenic acid (ALA)	0.31 (0.77)	0.33 (0.97)	0.3 (0.75)	0.3 (0.17)	0.309	
C20:5 (n3) Eicosapentenoic acid (EPA)	0.3 (0.27)	0.28 (0.19)	0.34 (0.29)	0.26 (0.33)	0.251	
C22:6 (n3) Docosahexenoic acid (DHA)	1.42 (0.92)	1.52 (0.85)	1.34 (0.97)	1.33 (0.87)	0.123	
C18:2 (n6) Linoleic acid (LA)	14.06 (4.12)	13.77 (3.98)	14.64 (4.43)	13.46 (3.4)	0.057	
C20:4 (n6) Arachidonic acid (AA)	5.04 (1.95)	5.18 (1.65)	5 (2.4)	4.75 (1.28)	0.150	
Plasma total *n*-3 PUFA	2.33 (1.53)	2.54 (1.29)	2.17 (1.78)	2.34 (1.35)	0.069	
Plasma total *n*-6 PUFA	18.92 (5.09)	18.85 (4.6)	19.11 (5.42)	18.64 (3.88)	0.247	
Plasma *n*-6/*n*-3 ratio	8.28 (5.85)	7.94 (4.57)	8.93 (8.14)	7.77 (6.58)	0.091	
Plasma total SFA	67.75 (7.45)	68.34 (6.93)	67.13 (8.58)	68.48 (5.22)	0.079	
Plasma total MUFA	10.9 (4.31)	10.42 (3.08) (a)	11.75 (4.75)	10.37 (3.65)	0.016	0.057
Plasma total PUFA	21.48 (4.43)	21.48 (3.78)	21.68 (5.05)	20.85 (3.76)	0.284	

Between-group comparisons were performed using the Kruskal Wallis test. (a) Poshoc analysis by Dunn’s test, *p* < 0.05 vs. osteopenia group. (b) Poshoc analysis by Dunn’s test, *p* < 0.05 vs. osteoporosis group * Adjusted by, age, years since menopause, BMI and vitamin D, calcium and energy intake (Quade’s test).

**Table 3:** Bone parameters by tertile of plasma *n*-3 PUFA (C18:3 (n3) Linolenic acid (ALA) + C20:5 (n3) Eicosapentenoic acid (EPA) + C22:6 (n3) Docosahexenoic acid (DHA)).

		Plasma Total *n*-3 PUFA Tertiles				
	Total Sample (*n* = 301)	Lowest (<1.91) (*n* = 98)	Middle (1.91–2.79) (*n* = 101)	Highest (>2.79) (*n* = 102)		
	Median (IQR); *n* (%)	Median (IQR); *n* (%)	Median (IQR); *n* (%)	Median (IQR); *n* (%)	*p* Value	Adjusted *p* Value *
Quantitative Bone Ultrasound						
BUA, dB/MHz	106 (14)	99 (10)	106 (15)	108 (14)	0.203	
SOS, m/s	1541 (35)	1526 (29)	1544 (38)	1541 (36)	0.531	
Volumetric BMD (mg/cm^3^)						
Total density (mg/cm^3^)	305.7 (73.7)	261.6 (71.5)	303 (81.4)	313.3 (80.3)	0.124	
Trabecular density (mg/cm^3^)	163.1 (55.4)	131.7 (54.7)	164.5 (51.6)	161.5 (62.9)	0.72	
Cortical density (mg/cm^3^)	418 (109.9)	350.3 (108.5) (a)	416 (123.7)	430.2 (98.9)	0.048	0.013
Bone morphometry (mm^2^)						
Total area (mm^2^)	300.4 (54)	272.2 (59.5)	300.4 (55.7)	301.8 (48.8)	0.373	
Trabecular area (mm^2^)	135.1 (24.4)	122.2 (26.8)	135.1 (25.1)	136.6 (24)	0.273	
Cortical area (mm^2^)	165.3 (29.9)	150 (32.8)	165.3 (30.6)	167.1 (29.2)	0.274	
Bone Mineral Density						
BMD L2-L4 (g/cm^2^)	0.912 (0.205)	0.815 (0.186) (a)	0.907 (0.216)	0.936 (0.232)	0.011	0.043
BMD L2 (g/cm^2^)	0.901 (0.204)	0.812 (0.171) (a)	0.902 (0.193)	0.933 (0.221)	0.023	0.123
BMD L3 (g/cm^2^)	0.925 (0.229)	0.821 (0.197) (a)	0.924 (0.243)	0.949 (0.226)	0.033	0.074
BMD L4 (g/cm^2^)	0.914 (0.204)	0.801 (0.184) (a)	0.895 (0.203)	0.954 (0.234)	0.004	0.021
Z-score (lumbar spine)	0.3 (1.6)	−0.6 (1.3) (a)	0.2 (1.7) (b)	0.6 (1.6)	0.001	0.034
T-score (lumbar spine)	−1.3 (2)	−2.3 (1.8) (a)	−1.3 (2)	−1.1 (2.2)	0.01	0.050
BMD Femoral trochanter (g/cm^2^)	0.759 (0.13)	0.704 (0.107)	0.753 (0.185)	0.786 (0.149)	0.239	
BMD Femoral neck (g/cm^2^)	0.611 (0.139)	0.529 (0.125) (a)	0.612 (0.136)	0.644 (0.138)	0.042	0.054
Z-score (hip)	0.5 (1.4)	−0.2 (1.1) (a)	0.4 (1.7)	0.8 (1.7)	0.035	0.064
T-score (hip)	−0.8 (1.3)	−1.3 (1.1)	−0.8 (1.7)	−0.5 (1.5)	0.234	
Bone health						
Normal	*n* = 103 (34.2%)	*n* = 24 (23.3%)	*n* = 38 (36.9%)	*n* = 41 (39.8%)	0.113	
Osteopenia	*n* = 145 (48.2%)	*n* = 55 (37.9%)	*n* = 43 (29.7%)	*n* = 47 (32.4%)		
Osteoporosis	*n* = 53 (17.6%)	*n* = 19 (35.8%)	*n* = 20 (37.7%)	*n* = 14 (26.4%)		

Between-group comparisons were performed using the Kruskal Wallis test or the Chi-square test as appropriate. (a) Poshoc analysis by Dunn’s test, *p* < 0.05 vs. higher tertile. (b) Poshoc analysis by Dunn’s test, *p* < 0.05 vs. higher tertile. * Adjusted by, age, years since menopause, BMI and vitamin D, calcium and energy intake (Quade’s test).

**Table 4:** Bone parameters by tertile of plasma *n*-6 PUFA (C18:2 (n6) Linoleic acid (LA) + C20:4 (n6) Arachidonic acid (AA)).

		Plasma Total *n*-6 PUFA Tertiles				
	Total Sample (*n* = 301)	Lowest (<17.59) (*n* = 98)	Middle (17.59–20.49) (*n* = 101)	Highest (>20.49) (*n* = 102)		
	Median (IQR); *n* (%)	Median (IQR); *n* (%)	Median (IQR); *n* (%)	Median (IQR); *n* (%)	*p* Value	Adjusted *p* Value *
Quantitative Bone Ultrasound						
BUA (dB/MHz)	106 (14)	105 (14)	106 (14)	107 (13)	0.599	
SOS (m/s)	1541 (35)	1540 (29)	1539 (36) (a)	1546 (35)	0.045	0.320
Volumetric BMD (mg/cm^3^)						
Total density (mg/cm^3^)	305.7 (73.7)	298.2 (82.4)	309.9 (74.7)	308 (65.9)	0.546	
Trabecular density (mg/cm^3^)	163.1 (55.4)	162.1 (55.3)	164.6 (62.3)	164 (51.7)	0.664	
Cortical density (mg/cm^3^)	418 (109.9)	409 (120.6)	429.3 (102.5)	423.1 (107.2)	0.422	
Bone morphometry (mm^2^)						
Total area (mm^2^)	300.4 (54)	299.5 (48.1)	304 (51.2)	294 (55)	0.123	
Trabecular area (mm^2^)	135.1 (24.4)	135.3 (21)	136.8 (22.9)	132.1 (24.8)	0.11	
Cortical area (mm^2^)	165.3 (29.9)	165.5 (26.1)	167 (28.2)	161.9 (30.3)	0.116	
Bone Mineral Density						
BMD L2-L4 (g/cm^2^)	0.912 (0.205)	0.913 (0.252)	0.92 (0.187)	0.903 (0.194)	0.828	
BMD L2 (g/cm^2^)	0.901 (0.204)	0.886 (0.221)	0.915 (0.196)	0.884 (0.185)	0.746	
BMD L3 (g/cm^2^)	0.925 (0.229)	0.906 (0.266)	0.942 (0.197)	0.915 (0.197)	0.759	
BMD L4 (g/cm^2^)	0.914 (0.204)	0.926 (0.258)	0.913 (0.186)	0.916 (0.178)	0.873	
Z-score (lumbar spine)	0.3 (1.6)	0.3 (2.2)	0.3 (1.3)	0.1 (1.5)	0.694	
T-score (lumbar spine)	−1.3 (2)	−1.3 (2.4)	−1.3 (1.8)	−1.4 (1.8)	0.856	
BMD Femoral trochanter (g/cm^2^)	0.759 (0.13)	0.756 (0.135)	0.756 (0.121)	0.762 (0.149)	0.855	
BMD Femoral neck (g/cm^2^)	0.611 (0.139)	0.607 (0.148)	0.622 (0.12)	0.606 (0.135)	0.645	
Z-score (hip)	0.5 (1.4)	0.6 (1.3)	0.5 (1.5)	0.5 (1.4)	0.95	
T-score (hip)	−0.8 (1.3)	−0.8 (1.2)	−0.8 (1.3)	−0.7 (1.4)	0.856	
Bone health						
Normal	*n* = 103 (34.2%)	*n* = 38 (36.9%)	*n* = 32 (31.1%)	*n* = 33 (32%)	0.390	
Osteopenia	*n* = 145 (48.2%)	*n* = 40 (27.6%)	*n* = 50 (34.5%)	*n* = 55 (37.9%)		
Osteoporosis	*n* = 53 (17.6%)	*n* = 20 (37.7%)	*n* = 19 (35.8%)	*n* = 14 (26.4%)		

Between-group comparisons were performed using the Kruskal Wallis test or the Chi-square test as appropriate. (a) Poshoc analysis by Dunn’s test, *p* < 0.05 vs. higher tertile. * Adjusted by, age, years since menopause, BMI and vitamin D, calcium and energy intake (Quade’s test).

**Table 5:** Bone parameters by tertile of plasma *n*-6 PUFA/*n*-3 PUFA ratio.

		Plasma Total *n*-6/*n*-3 PUFA Tertiles				
	Total Sample (*n* = 301)	Lowest (<6.79) (*n* = 98)	Middle (6.79–10.27) (*n* = 101)	Highest (>10.27) (*n* = 102)		
	Median (IQR); *n* (%)	Median (IQR); *n* (%)	Median (IQR); *n* (%)	Median (IQR); *n* (%)	*p* Value	Adjusted *p* Value *
Quantitative Bone Ultrasound						
BUA (dB/MHz)	106 (14)	105 (14)	106 (14)	107 (13)	0.683	
SOS (m/s)	1541 (35)	1540 (29)	1539 (36)	1546 (35)	0.982	
Volumetric BMD (mg/cm^3^)						
Total density (mg/cm^3^)	305.7 (73.7)	298.2 (82.4)	309.9 (74.7)	308 (65.9)	0.154	
Trabecular density (mg/cm^3^)	163.1 (55.4)	162.1 (55.3)	164.6 (62.3)	164 (51.7)	0.875	
Cortical density (mg/cm^3^)	418 (109.9)	409 (120.6)	429.3 (102.5)	423.1 (107.2)	0.099	
Bone morphometry (mm^2^)						
Total area (mm^2^)	300.4 (54)	299.5 (48.1)	304 (51.2)	294 (55)	0.966	
Trabecular area (mm^2^)	135.1 (24.4)	135.3 (21)	136.8 (22.9)	132.1 (24.8)	0.88	
Cortical area (mm^2^)	165.3 (29.9)	165.5 (26.1)	167 (28.2)	161.9 (30.3)	0.883	
Bone Mineral Density						
BMD L2-L4 (g/cm^2^)	0.912 (0.205)	0.913 (0.252)	0.92 (0.187)	0.903 (0.194)	0.152	
BMD L2 (g/cm^2^)	0.901 (0.204)	0.886 (0.221)	0.915 (0.196)	0.884 (0.185)	0.247	
BMD L3 (g/cm^2^)	0.925 (0.229)	0.906 (0.266)	0.942 (0.197)	0.915 (0.197)	0.3	
BMD L4 (g/cm^2^)	0.914 (0.204)	0.926 (0.258)	0.913 (0.186)	0.916 (0.178)	0.05	
Z-score (lumbar spine)	0.3 (1.6)	0.3 (2.2) (a)	0.3 (1.3) (b)	0.1 (1.5)	0.016	0.166
T-score (lumbar spine)	−1.3 (2)	−1.3 (2.4)	−1.3 (1.8)	−1.4 (1.8)	0.132	
BMD Femoral trochanter (g/cm^2^)	0.759 (0.13)	0.756 (0.135)	0.756 (0.121)	0.762 (0.149)	0.48	
BMD Femoral neck (g/cm^2^)	0.611 (0.139)	0.607 (0.148)	0.622 (0.12)	0.606 (0.135)	0.157	
Z-score (hip)	0.5 (1.4)	0.6 (1.3)	0.5 (1.5)	0.5 (1.4)	0.081	
T-score (hip)	−0.8 (1.3)	−0.8 (1.2)	−0.8 (1.3)	−0.7 (1.4)	0.413	
Bone health						
Normal	*n* = 103 (34.2%)	*n* = 38 (36.9%)	*n* = 39 (37.9%)	*n* = 26 (25.2%)	0.194	
Osteopenia	*n* = 145 (48.2%)	*n* = 42 (29%)	*n* = 46 (31.7%)	*n* = 57 (39.3%)		
Osteoporosis	*n* = 53 (17.6%)	*n* = 19 (35.8%)	*n* = 15 (28.3%)	*n* = 19 (25.8%)		

Between-group comparisons were performed using the Kruskal Wallis test or the Chi-square test as appropriate. (a) Poshoc analysis by Dunn’s test, *p* < 0.05 vs. higher tertile. (b) Poshoc analysis by Dunn’s test, *p* < 0.05 vs. higher tertile. * Adjusted by, age, years since menopause, BMI and vitamin D, calcium and energy intakes (Quade’s test).

**Table 6:** Bone parameters by tertile of plasma Saturated Fatty Acids (SFA).

		Plasma Total SFA Tertiles			
	Total Sample (*n* = 301)	Lowest (<65.35) (*n* = 99)	Middle (65.35–69.28) (*n* = 100)	Highest (>69.28) (*n* = 102)	
	Median (IQR); *n* (%)	Median (IQR); *n* (%)	Median (IQR); *n* (%)	Median (IQR); *n* (%)	*p* Value
Quantitative Bone Ultrasound					
BUA (dB/MHz)	106 (14)	107 (12)	106 (13)	105 (16)	0.604
SOS (m/s)	1541 (35)	1549 (35)	1540 (31)	1540 (32)	0.052
Volumetric BMD (mg/cm^3^)					
Total density (mg/cm^3^)	305.7 (73.7)	302.4 (68.4)	303.2 (71.5)	308 (80.7)	0.726
Trabecular density (mg/cm^3^)	163.1 (55.4)	163.4 (50)	162.4 (60.8)	164.3 (60.6)	0.902
Cortical density (mg/cm^3^)	418 (109.9)	414 (109.7)	419 (109.4)	426.5 (117.4)	0.653
Bone morphometry (mm^2^)					
Total area (mm^2^)	300.4 (54)	296.9 (48.4)	303.1 (53.6)	302 (53.2)	0.154
Trabecular area (mm^2^)	135.1 (24.4)	133 (21.6)	136.4 (24.2)	136 (22.2)	0.135
Cortical area (mm^2^)	165.3 (29.9)	163.6 (26.8)	166.7 (29.4)	166.6 (27.5)	0.142
Bone Mineral Density					
BMD L2-L4 (g/cm^2^)	0.912 (0.205)	0.901 (0.18)	0.917 (0.204)	0.926 (0.225)	0.738
BMD L2 (g/cm^2^)	0.901 (0.204)	0.88 (0.186)	0.894 (0.188)	0.922 (0.213)	0.862
BMD L3 (g/cm^2^)	0.925 (0.229)	0.913 (0.204)	0.925 (0.225)	0.946 (0.241)	0.776
BMD L4 (g/cm^2^)	0.914 (0.204)	0.896 (0.171)	0.919 (0.223)	0.924 (0.225)	0.709
Z-score (lumbar spine)	0.3 (1.6)	0.1 (1.4)	0.3 (1.5)	0.4 (1.9)	0.697
T-score (lumbar spine)	−1.3 (2)	−1.4 (1.7)	−1.3 (1.9)	−1.2 (2.2)	0.727
BMD Femoral trochanter (g/cm^2^)	0.759 (0.13)	0.756 (0.157)	0.757 (0.121)	0.771 (0.149)	0.715
BMD Femoral neck (g/cm^2^)	0.611 (0.139)	0.607 (0.135)	0.609 (0.137)	0.625 (0.145)	0.486
Z-score (hip)	0.5 (1.4)	0.5 (1.6)	0.5 (1.2)	0.5 (1.5)	0.751
T-score (hip)	−0.8 (1.3)	−0.8 (1.4)	−0.7 (1.1)	−0.7 (1.6)	0.664
Bone health					
Normal	*n* = 103 (34.2%)	*n* = 29 (28.2%)	*n* = 33 (32%)	*n* = 41 (39.8%)	0.100
Osteopenia	*n* = 145 (48.2%)	*n* = 57 (39.3%)	*n* = 49 (33.8%)	*n* = 39 (26.9%)	
Osteoporosis	*n* = 53 (17.6%)	*n* = 13 (24.5%)	*n* = 18 (34%)	*n* = 22 (41.5%)	

Between-group comparisons were performed using the Kruskal Wallis test or the Chi-square test as appropriate. SFA (Saturated Fatty Acid: C8:0 Caprylic acid, + C10:0 Decanoic acid, + C12:0 Lauric acid, + C14:0 Myristic acid, + C16:0 Palmitic acid, + C18:0 Stearic acid, + C20:0 Arachidic acid, + C22:0 Docosanoic acid, + C24:0 Lignoceric acid).

**Table 7:** Bone parameters by tertile of plasma Monounsaturated Fatty Acids (MUFA) (C16:1 Palmitoleic acid + C18:1 cis (n9) Oleic acid + C22:1 (n9) Erucic acid).

		Plasma Total MUFA Tertiles				
	Total Sample (*n* = 301)	Lowest (<9.66) (*n* = 99)	Middle (9.66–12.37) (*n* = 100)	Highest (>12.37) (*n* = 102)		
	Median (IQR); *n* (%)	Median (IQR); *n* (%)	Median (IQR); *n* (%)	Median (IQR); *n* (%)	*p* Value	Adjusted *p* Value *
Quantitative Bone Ultrasound						
BUA (dB/MHz)	106 (14)	107 (17)	105 (12)	106 (11)	0.395	
SOS (m/s)	1541 (35)	1540 (33)	1541 (38)	1544 (35)	0.836	
Volumetric BMD (mg/cm^3^)						
Total density (mg/cm^3^)	305.7 (73.7)	309.4 (79.9)	306.7 (83)	294.7 (69.1)	0.409	
Trabecular density (mg/cm^3^)	163.1 (55.4)	159.1 (59.4)	164.6 (57.8)	164.5 (53.6)	0.885	
Cortical density (mg/cm^3^)	418 (109.9)	434.1 (109.2)	420.6 (121.5)	408.5 (106.5)	0.141	
Bone morphometry (mm^2^)						
Total area (mm^2^)	300.4 (54)	307 (48.8) (a)	302.2 (63.9) (b)	290.7 (41.8)	0.02	0.012
Trabecular area (mm^2^)	135.1 (24.4)	137.8 (21.2) (a)	135.8 (28.5) (b)	130.9 (18.8)	0.014	0.006
Cortical area (mm^2^)	165.3 (29.9)	169.2 (25.8) (a)	166.4 (35.4) (b)	160.3 (23)	0.014	0.006
Bone Mineral Density						
BMD L2-L4 (g/cm^2^)	0.912 (0.205)	0.927 (0.216)	0.934 (0.228)	0.883 (0.173)	0.078	
BMD L2 (g/cm^2^)	0.901 (0.204)	0.919 (0.22)	0.912 (0.226)	0.865 (0.161)	0.07	
BMD L3 (g/cm^2^)	0.925 (0.229)	0.941 (0.214)	0.953 (0.245)	0.896 (0.162)	0.056	
BMD L4 (g/cm^2^)	0.914 (0.204)	0.933 (0.205)	0.923 (0.231)	0.879 (0.179)	0.26	
Z-score (lumbar spine)	0.3 (1.6)	0.4 (1.9)	0.3 (1.6)	0 (1.4)	0.308	
T-score (lumbar spine)	−1.3 (2)	−1.2 (2)	−1.1 (2.2)	−1.6 (1.7)	0.075	
BMD Femoral trochanter (g/cm^2^)	0.759 (0.13)	0.779 (0.171)	0.759 (0.127)	0.744 (0.139)	0.219	
BMD Femoral neck (g/cm^2^)	0.611 (0.139)	0.63 (0.135)	0.594 (0.143)	0.609 (0.138)	0.229	
Z-score (hip)	0.5 (1.4)	0.6 (1.7)	0.5 (1.3)	0.4 (1.4)	0.586	
T-score (hip)	−0.8 (1.3)	−0.6 (1.7)	−0.7 (1.2)	−0.9 (1.3)	0.179	
Bone health						
Normal	*n* = 103 (34.2%)	*n* = 37 (35.9%)	*n* = 42 (40.8%)	*n* = 24 (23.3%)	0.020	
Osteopenia	*n* = 145 (48.2%)	*n* = 41 (28.3%)	*n* = 42 (29%)	*n* = 62 (42.8%)		
Osteoporosis	*n* = 53 (17.6%)	*n* = 21 (39.6%)	*n* = 16 (30.2%)	*n* = 16 (30.2%)		

Between-group comparisons were performed using the Kruskal Wallis test or the Chi-square test as appropriate. (a) Poshoc analysis by Dunn’s test, *p* < 0.05 vs. higher tertile. (b) Poshoc analysis by Dunn’s test, *p* < 0.05 vs. higher tertile. * Adjusted by, age, years since menopause, BMI and vitamin D, calcium and energy intakes (Quade’s test).

**Table 8:** Bone parameters by tertile of total plasma PUFA.

		Plasma Total PUFA Tertiles				
	Total Sample (*n* = 301)	Lowest (<20.28) (*n* = 99)	Middle (20.28–22.95) (*n* = 101)	Highest (>22.95) (*n* = 101)		
	Median (IQR); *n* (%)	Median (IQR); *n* (%)	Median (IQR); *n* (%)	Median (IQR); *n* (%)	*p* Value	Adjusted *p* Value *
Quantitative Bone Ultrasound						
BUA (dB/MHz)	106 (14)	105 (15)	106 (15)	107 (14)	0.125	
SOS (m/s)	1541 (35)	1538 (28) (a)	1541 (31) (b)	1551 (38)	0.004	0.588
Volumetric BMD (mg/cm^3^)						
Total density (mg/cm^3^)	305.7 (73.7)	303.2 (82.3)	307.3 (80.3)	306.3 (65.70)	0.671	
Trabecular density (mg/cm^3^)	163.1 (55.4)	162.2 (59.6)	166.5 (57.1)	161.4 (51.5)	0.49	
Cortical density (mg/cm^3^)	418 (109.9)	414.2 (124.8)	429.3 (103.5)	421.5 (108.8)	0.517	
Bone morphometry (mm^2^)						
Total area (mm^2^)	300.4 (54)	296.6 (47.4)	302.8 (53.3)	298 (57.8)	0.512	
Trabecular area (mm^2^)	135.1 (24.4)	133.3 (21.2)	136.8 (24)	134 (26.1)	0.405	
Cortical area (mm^2^)	165.3 (29.9)	163.3 (26.3)	167 (28.9)	164.7 (31.7)	0.414	
Bone Mineral Density						
BMD L2-L4 (g/cm^2^)	0.912 (0.205)	0.883 (0.215)	0.934 (0.185)	0.904 (0.198)	0.14	
BMD L2 (g/cm^2^)	0.901 (0.204)	0.873 (0.209)	0.923 (0.211)	0.901 (0.185)	0.216	
BMD L3 (g/cm^2^)	0.925 (0.229)	0.898 (0.251)	0.951 (0.206)	0.916 (0.21)	0.144	
BMD L4 (g/cm^2^)	0.914 (0.204)	0.877 (0.244)	0.939 (0.182)	0.914 (0.181)	0.094	
Z-score (lumbar spine)	0.3 (1.6)	0.1 (2)	0.4 (1.2)	0.2 (1.4)	0.305	
T-score (lumbar spine)	−1.3 (2)	−1.6 (2)	−1.1 (1.8)	−1.4 (1.9)	0.173	
BMD Femoral trochanter (g/cm^2^)	0.759 (0.13)	0.745 (0.13)	0.78 (0.103)	0.759 (0.177)	0.178	
BMD Femoral neck (g/cm^2^)	0.611 (0.139)	0.602 (0.137)	0.625 (0.118)	0.604 (0.141)	0.291	
Z-score (hip)	0.5 (1.4)	0.4 (1.4)	0.6 (1.4)	0.5 (1.6)	0.313	
T-score (hip)	−0.8 (1.3)	−0.9 (1.3)	−0.6 (1.1)	−0.8 (1.6)	0.197	
Bone health						
Normal	*n* = 103 (34.2%)	*n* = 31 (30.1)	*n* = 39 (37.9%)	*n* = 33 (32.0%)	0.428	
Osteopenia	*n* = 145 (48.2%)	*n* = 46 (31.7%)	*n* = 45 (31%)	*n* = 54 (37.2%)		
Osteoporosis	*n* = 53 (17.6%)	*n* = 22 (41.5%)	*n* = 17 (32.1%)	*n* = 14 (26.4%)		

Between-group comparisons were performed using the Kruskal Wallis test or the Chi-square test as appropriate. (a) Poshoc analysis by Dunn’s test, *p* < 0.05 vs. higher tertile. (b) Poshoc analysis by Dunn’s test, *p* < 0.05 vs. higher tertile. * Adjusted by, age, years since menopause, BMI and vitamin D, calcium and energy intakes (Quade’s test). Total plasma PUFA: C18:3 (n3) Linolenic acid (ALA) + C20:5 (n3) Eicosapentenoic acid (EPA) + C22:6 (n3) Docosahexenoic acid (DHA) + C18:2 (n6) Linoleic acid (LA) + C20:4 (n6) Arachidonic acid (AA).

**Table 9:** Bivariate and partial correlations between plasma fatty acids and bone mineral density at either the lumbar spine or the hips.

Variables		BMD L2-L4 (g/cm^2^)	BMD L2 (g/cm^2^)	BMD L3 (g/cm^2^)	BMD L4 (g/cm^2^)	BMD Femoral Trochanter (g/cm^2^)	BMD Femoral Neck (g/cm^2^)
Plasma total *n*-3 PUFA	Spearman Rho	0.157	0.143	0.128	0.178	0.11	0.153
	*p* Value	0.006	0.013	0.026	0.002	0.056	0.008
	Adjusted coefficient *	0.150	0.141	0.129	0.170		0.143
	*p* Value	0.014	0.021	0.035	0.005		0.019
Plasma total *n*-6 PUFA	Spearman Rho	0.015	0.041	0.026	−0.013	0.027	−0.021
	*p* Value	0.802	0.481	0.652	0.819	0.636	0.721
Plasma *n*-6/*n*-3 ratio	Spearman Rho	−0.121	−0.094	−0.094	−0.156	−0.069	−0.127
	*p* Value	0.035	0.102	0.102	0.007	0.23	0.035
	Adjusted coefficient *	−0.105			−0.139		−0.110
	*p* Value	0.086			0.023		0.073
Plasma total SFA	Spearman Rho	0.031	0.016	0.026	0.034	0.027	0.057
	*p* Value	0.59	0.776	0.659	0.552	0.639	0.324
Plasma total MUFA	Spearman Rho	−0.106	−0.113	−0.097	−0.082	−0.101	−0.091
	*p* Value	0.066	0.05	0.092	0.155	0.08	0.114
Plasma total PUFA	Spearman Rho	0.056	0.077	0.058	0.037	0.056	0.012
	*p* Value	0.336	0.18	0.312	0.517	0.33	0.829

* Partial non parametric correlations adjusted by, age, years since menopause, BMI and vitamin D, calcium and energy intakes.

**Table 10:** Multiple linear regression analysis for the BMD at the lumbar spine.

*Total sample. Spine BMD*				
Optimal model	R2	Adjusted R2	F	*p*
	0.126	0.116	12.552	<0.001
Selected independent variables		standardized B	t	*p*
BMI (kg/m^2^)		0.288	4.961	<0.001
Age (years)		−0.163	−2.769	0.006
Plasma total *n*-3 PUFA		0.155	2.643	0.009
*Normal women. Spine BMD*				
Optimal model	R2	Adjusted R2	F	*p*
	0.074	0.064	6.922	0.01
Selected independent variables		standardized B	t	*p*
Plasma total *n*-3 PUFA		0.273	2.631	0.01
*Osteopenic women. Spine BMD*				
Optimal model	R2	Adjusted R2	F	*p*
	0.051	0.044	6.737	0.011
Selected independent variables		standardized B	t	*p*
Energy (kcal/day)		0.226	2.596	0.011
*Osteoporotic women. Spine BMD*				
Optimal model	R2	Adjusted R2	F	*p*
	0.283	0.253	9.282	<0.001
Selected independent variables		standardized B	t	*p*
Plasma total MUFA		−0.411	−3.303	0.002
Plasma total *n*-3 PUFA		0.290	2.323	0.024

Predictors: Age (years), Years since menopause (years), BMI (kg/m^2^), Vitamin D (µg/day), Calcium intake (mg/day), Energy (kcal/day), Plasma total *n*-3 PUFA, Plasma total *n*-6 PUFA, Plasma *n*-6/*n*-3 ratio, Plasma total SFA, Plasma total MUFA and Plasma total PUFA.

**Table 11:** Multiple linear regression analysis for the BMD at the femoral neck.

*Total sample. Femoral neck BMD*				
Optimal model	R2	Adjusted R2	F	*p*
	0.185	0.179	29.946	<0.001
Selected independent variables		standardized B	t	*p*
BMI (kg/m^2^)		0.394	7.068	<0.001
Plasma total *n*-3 PUFA		0.146	2.615	0.009
*Normal women sample. Femoral neck BMD*				
Optimal model	R2	Adjusted R2	F	*p*
	0.187	0.168	9.797	<0.001
Selected independent variables		standardized B	t	*p*
BMI (kg/m^2^)		0.320	3.227	0.002
Plasma total *n*-3 PUFA		0.245	2.477	0.015
*Osteopenic women sample. Femoral neck BMD*				
Optimal model	R2	Adjusted R2	F	*p*
	0.090	0.082	12.410	0.001
Selected independent variables		standardized B	t	*p*
BMI (kg/m^2^)		0.299	3.523	0.001

Predictors: Age, years. Years since menopause, years. BMI (kg/m^2^), Vitamin D (µg/day), Calcium intake (mg/day), Energy (kcal/day), Plasma total *n*-3 PUFA, Plasma total *n*-6 PUFA, Plasma *n*-6/*n*-3 ratio, Plasma total SFA, Plasma total MUFA and Plasma total PUFA.

**Table 12:** Logistic regression of predictors associated with low BMD (T ≤ −1 score).

	Univariate			Multivariate		
	OR	95% CI	*p* Value	OR	95% CI	*p* Value
Age, years	1.040	0.997–1.084	0.068			
Years since menopause, years	0.987	0.956–1.020	0.439			
BMI (kg/m^2^)	0.905	0.858–0.954	<0.001	0.893	0.841–0.948	*p* < 0.001
Vitamin D (µg/day)	1.000	0.993–1.006	0.895			
Calcium intake (mg/day)	1.000	1.000–1.001	0.785			
Energy (kcal/day)	1.000	1.000–1.000	0.676			
Plasma total *n*-3 PUFA	0.791	0.635–0.984	0.035	0.751	0.587–0.960	0.022
Plasma total *n*-6 PUFA	1.014	0.953–1.079	0.655			
Plasma *n*-6/*n*-3 ratio	1.024	0.998–1.050	0.069			
Plasma total SFA	0.982	0.942–1.023	0.379			
Plasma total MUFA	1.073	0.993–1.158	0.075			
Plasma total PUFA.	0.995	0.935–1.058	0.874			

OR: Odds ratio; CI: Confidence interval; Reference level_ T-score at either spine or femoral neck > −1. Multivariate model adjusted by: Age (years), Years since menopause (years) BMI (kg/m^2^), Vitamin D (µg/day), Calcium intake (mg/day), Energy (kcal/day).

## Data Availability

The dataset analyzed during the current study is not publicly available due to national data regulations and for ethical reasons, including that we do not have the explicit written consent of the study volunteers to make their deidentified data available at the end of the study. However, datasets and SPSS statistical analyses can be requested by sending a letter to the corresponding author.

## Supplementary Materials

The following are available online at https://www.mdpi.com/article/10.3390/nu13051454/s1: Table S1: Bivariate and partial correlations between plasma fatty acid profile and bone mineral density in the lumbar spine and the hips; Table S2: Logistic regression of fatty acids associated with low BMD (T-score ≤ −1).

## References

[1] Weiss L.A., Barrett-Connor E., von Mühlen D. (2005). Ratio of n–6 to n–3 Fatty Acids and Bone Mineral Density in Older Adults: The Rancho Bernardo Study. Am. J. Clin. Nutr..

[2] Eriksson S., Mellström D., Strandvik B. (2009). Fatty Acid Pattern in Serum Is Associated with Bone Mineralisation in Healthy 8-Year-Old Children. Br. J. Nutr..

[3] Hogstrom M., Nordström P., Nordström A. (2007). N-3 Fatty Acids Are Positively Associated with Peak Bone Mineral Density and Bone Accrual in Healthy Men: The NO_2_ Study. Am. J. Clin. Nutr..

[4] Sánchez-Borrego R., von Schacky C., Osorio M.J.A., Llaneza P., Pinto X., Losa F., Navarro M.C., Lubián D., Mendoza N. (2017). Recommendations of the Spanish Menopause Society on the Consumption of Omega-3 Polyunsaturated Fatty Acids by Postmenopausal Women. Maturitas.

[5] Farina E.K., Kiel D.P., Roubenoff R., Schaefer E.J., Cupples L.A., Tucker K.L. (2012). Plasma Phosphatidylcholine Concentrations of Polyunsaturated Fatty Acids Are Differentially Associated With Hip Bone Mineral Density and Hip Fracture in Older Adults: The Framingham Osteoporosis Study. J. Bone Miner. Res..

[6] Williams C.M., Burdge G. (2006). Long-Chain *n*-3 PUFA: Plant v. Marine Sources. Proc. Nutr. Soc..

[7] Nawata K., Yamauchi M., Takaoka S., Yamaguchi T., Sugimoto T. (2013). Association of N-3 Polyunsaturated Fatty Acid Intake with Bone Mineral Density in Postmenopausal Women. Calcif. Tissue Int..

[8] Järvinen R., Tuppurainen M., Erkkilä A.T., Penttinen P., Kärkkäinen M., Salovaara K., Jurvelin J.S., Kröger H. (2012). Associations of Dietary Polyunsaturated Fatty Acids with Bone Mineral Density in Elderly Women. Eur. J. Clin. Nutr..

[9] Mangano K.M., Sahni S., Kerstetter J.E., Kenny A.M., Hannan M.T. (2013). Polyunsaturated Fatty Acids and Their Relation with Bone and Muscle Health in Adults. Curr. Osteoporos. Rep..

[10] Haag M., Magada O.N., Claassen N., Böhmer L.H., Kruger M.C. (2003). Omega-3 Fatty Acids Modulate ATPases Involved in Duodenal Ca Absorption. Prostaglandins Leukot. Essent. Fat. Acids.

[11] Casado-Díaz A., Santiago-Mora R., Dorado G., Quesada-Gómez J.M. (2013). The Omega-6 Arachidonic Fatty Acid, but Not the Omega-3 Fatty Acids, Inhibits Osteoblastogenesis and Induces Adipogenesis of Human Mesenchymal Stem Cells: Potential Implication in Osteoporosis. Osteoporos. Int..

[12] Coetzee M., Haag M., Joubert A.M., Kruger M.C. (2007). Effects of Arachidonic Acid, Docosahexaenoic Acid and Prostaglandin E2 on Cell Proliferation and Morphology of MG-63 and MC3T3-E1 Osteoblast-like Cells. Prostaglandins Leukot. Essent. Fat. Acids.

[13] Zwart S.R., Pierson D., Mehta S., Gonda S., Smith S.M. (2010). Capacity of Omega-3 Fatty Acids or Eicosapentaenoic Acid to Counteract Weightlessness-Induced Bone Loss by Inhibiting NF-ΚB Activation: From Cells to Bed Rest to Astronauts. J. Bone Miner. Res..

[14] Benetou V., Orfanos P., Zylis D., Sieri S., Contiero P., Tumino R., Giurdanella M.C., Peeters P.H.M., Linseisen J., Nieters A. (2011). Diet and Hip Fractures among Elderly Europeans in the EPIC Cohort. Eur. J. Clin. Nutr..

[15] Virtanen J.K., Mozaffarian D., Willett W.C., Feskanich D. (2012). Dietary Intake of Polyunsaturated Fatty Acids and Risk of Hip Fracture in Men and Women. Osteoporos. Int..

[16] Bao M., Zhang K., Wei Y., Hua W., Gao Y., Li X., Ye L. (2020). Therapeutic Potentials and Modulatory Mechanisms of Fatty Acids in Bone. Cell Prolif..

[17] Orchard T.S., Cauley J.A., Frank G.C., Neuhouser M.L., Robinson J.G., Snetselaar L., Tylavsky F., Wactawski-Wende J., Young A.M., Lu B. (2010). Fatty Acid Consumption and Risk of Fracture in the Women’s Health Initiative. Am. J. Clin. Nutr..

[18] Orchard T.S., Pan X., Cheek F., Ing S.W., Jackson R.D. (2012). A Systematic Review of Omega-3 Fatty Acids and Osteoporosis. Br. J. Nutr..

[19] Farina E.K., Kiel D.P., Roubenoff R., Schaefer E.J., Cupples L.A., Tucker K.L. (2011). Protective Effects of Fish Intake and Interactive Effects of Long-Chain Polyunsaturated Fatty Acid Intakes on Hip Bone Mineral Density in Older Adults: The Framingham Osteoporosis Study123. Am. J. Clin. Nutr..

[20] Lavado-García J., Roncero-Martin R., Moran J.M., Pedrera-Canal M., Aliaga I., Leal-Hernandez O., Rico-Martin S., Canal-Macias M.L. (2018). Long-Chain Omega-3 Polyunsaturated Fatty Acid Dietary Intake is Positively Associated with Bone Mineral Density in Normal and Osteopenic Spanish Women. PLoS ONE.

[21] Yuan S., Lemming E.W., Michaëlsson K., Larsson S.C. (2020). Plasma Phospholipid Fatty Acids, Bone Mineral Density and Fracture Risk: Evidence from a Mendelian Randomization Study. Clin. Nutr..

[22] Takkunen M., Ågren J., Kuusisto J., Laakso M., Uusitupa M., Schwab U. (2013). Dietary Fat in Relation to Erythrocyte Fatty Acid Composition in Men. Lipids.

[23] Patel P.S., Sharp S.J., Jansen E., Luben R.N., Khaw K.-T., Wareham N.J., Forouhi N.G. (2010). Fatty Acids Measured in Plasma and Erythrocyte-Membrane Phospholipids and Derived by Food-Frequency Questionnaire and the Risk of New-Onset Type 2 Diabetes: A Pilot Study in the European Prospective Investigation into Cancer and Nutrition (EPIC)–Norfolk Cohort. Am. J. Clin. Nutr..

[24] Sun Q., Ma J., Campos H., Hankinson S.E., Hu F.B. (2007). Comparison between Plasma and Erythrocyte Fatty Acid Content as Biomarkers of Fatty Acid Intake in US Women. Am. J. Clin. Nutr..

[25] Astorg P., Bertrais S., Laporte F., Arnault N., Estaquio C., Galan P., Favier A., Hercberg S. (2008). Plasma N-6 and *n*-3 Polyunsaturated Fatty Acids as Biomarkers of Their Dietary Intakes: A Cross-Sectional Study within a Cohort of Middle-Aged French Men and Women. Eur. J. Clin. Nutr..

[26] Lavado-Garcia J.M., Calderon-Garcia J.F., Moran J.M., Canal-Macias M.L., Rodriguez-Dominguez T., Pedrera-Zamorano J.D. (2012). Bone Mass of Spanish School Children: Impact of Anthropometric, Dietary and Body Composition Factors. J. Bone Miner. Metab..

[27] Moreiras O., Carbajal A., Cabrera L., Cuadrado C. (2013). Tablas de Composición de Alimentos.

[28] Templeton G. (2011). A Two-Step Approach for Transforming Continuous Variables to Normal: Implications and Recommendations for IS Research. Commun. Assoc. Inf. Syst..

[29] Svensson M., Schmidt E.B., Jørgensen K.A., Christensen J.H. (2008). The Effect of N-3 Fatty Acids on Lipids and Lipoproteins in Patients Treated with Chronic Haemodialysis: A Randomized Placebo-Controlled Intervention Study. Nephrol. Dial. Transplant..

[30] Kuroda T., Ohta H., Onoe Y., Tsugawa N., Shiraki M. (2017). Intake of Omega-3 Fatty Acids Contributes to Bone Mineral Density at the Hip in a Younger Japanese Female Population. Osteoporos. Int..

[31] Longo A.B., Ward W.E. (2016). PUFAs, Bone Mineral Density, and Fragility Fracture: Findings from Human Studies. Adv. Nutr..

[32] Sahni S., Mangano K.M., McLean R.R., Hannan M.T., Kiel D.P. (2015). Dietary Approaches for Bone Health: Lessons from the Framingham Osteoporosis Study. Curr. Osteoporos. Rep..

[33] Bellissimo M.P., Ziegler T.R., Jones D.P., Liu K.H., Fernandes J., Roberts J.L., Weitzmann M.N., Pacifici R., Alvarez J.A. (2020). Plasma High-Resolution Metabolomics Identifies Linoleic Acid and Linked Metabolic Pathways Associated with Bone Mineral Density. Clin. Nutr..

[34] Calderon-Garcia J.F., Moran J.M., Roncero-Martin R., Rey-Sanchez P., Rodriguez-Velasco F.J., Pedrera-Zamorano J.D. (2012). Dietary Habits, Nutrients and Bone Mass in Spanish Premenopausal Women: The Contribution of Fish to Better Bone Health. Nutrients.

[35] Harris T.B., Song X., Reinders I., Lang T.F., Garcia M.E., Siggeirsdottir K., Sigurdsson S., Gudnason V., Eiriksdottir G., Sigurdsson G. (2015). Plasma Phospholipid Fatty Acids and Fish-Oil Consumption in Relation to Osteoporotic Fracture Risk in Older Adults: The Age, Gene/Environment Susceptibility Study. Am. J. Clin. Nutr..

[36] Chen J.S., Hill C.L., Lester S., Ruediger C.D., Battersby R., Jones G., Cleland L.G., March L.M. (2016). Supplementation with Omega-3 Fish Oil Has No Effect on Bone Mineral Density in Adults with Knee Osteoarthritis: A 2-Year Randomized Controlled Trial. Osteoporos. Int..

[37] Watkins B.A., Li Y., Seifert M.F. (2006). Dietary Ratio of N-6/*n*-3 PUFAs and Docosahexaenoic Acid: Actions on Bone Mineral and Serum Biomarkers in Ovariectomized Rats. J. Nutr. Biochem..

[38] Simopoulos A.P. (2008). The Importance of the Omega-6/Omega-3 Fatty Acid Ratio in Cardiovascular Disease and Other Chronic Diseases. Exp. Biol. Med..

[39] Orchard T.S., Ing S.W., Lu B., Belury M.A., Johnson K., Wactawski-Wende J., Jackson R.D. (2013). The Association of Red Blood Cell N-3 and *n*-6 Fatty Acids with Bone Mineral Density and Hip Fracture Risk in the Women’s Health Initiative. J. Bone Miner. Res..

[40] Moon H.-J., Kim T.-H., Byun D.-W., Park Y. (2012). Positive Correlation between Erythrocyte Levels of N-3 Polyunsaturated Fatty Acids and Bone Mass in Postmenopausal Korean Women with Osteoporosis. Ann. Nutr. Metab..

[41] Mei Z., Dong X., Qian Y., Hong D., Xie Z., Yao G., Qin A., Gao S., Hu J., Liang L. (2020). Association between the Metabolome and Bone Mineral Density in a Chinese Population. EBioMedicine.

[42] Rozner R., Vernikov J., Griess-Fishheimer S., Travinsky T., Penn S., Schwartz B., Mesilati-Stahy R., Argov-Argaman N., Shahar R., Monsonego-Ornan E. (2020). The Role of Omega-3 Polyunsaturated Fatty Acids from Different Sources in Bone Development. Nutrients.

[43] Abou-Saleh H., Ouhtit A., Halade G.V., Rahman M.M. (2019). Bone Benefits of Fish Oil Supplementation Depend on Its EPA and DHA Content. Nutrients.

[44] Watkins B.A., Li Y., Seifert M.F. (2001). Nutraceutical Fatty Acids as Biochemical and Molecular Modulators of Skeletal Biology. J. Am. Coll. Nutr..

[45] Watkins B.A., Lippman H.E., Le Bouteiller L., Li Y., Seifert M.F. (2001). Bioactive Fatty Acids: Role in Bone Biology and Bone Cell Function. Prog. Lipid. Res..

[46] Loi F., Córdova L.A., Pajarinen J., Lin T., Yao Z., Goodman S.B. (2016). Inflammation, Fracture and Bone Repair. Bone.

[47] Li M., Ke H.Z., Qi H., Healy D.R., Li Y., Crawford D.T., Paralkar V.M., Owen T.A., Cameron K.O., Lefker B.A. (2003). A Novel, Non-Prostanoid EP2 Receptor-Selective Prostaglandin E2 Agonist Stimulates Local Bone Formation and Enhances Fracture Healing. J. Bone Miner. Res..

[48] Yang S., Feskanich D., Willett W.C., Eliassen A.H., Wu T. (2014). Association between Global Biomarkers of Oxidative Stress and Hip Fracture in Postmenopausal Women: A Prospective Study. J. Bone Miner. Res..

[49] Manolagas S.C., Parfitt A.M. (2010). What Old Means to Bone. Trends. Endocrinol. Metab..

[50] Zhao G., Etherton T.D., Martin K.R., Gillies P.J., West S.G., Kris-Etherton P.M. (2007). Dietary Alpha-Linolenic Acid Inhibits Proinflammatory Cytokine Production by Peripheral Blood Mononuclear Cells in Hypercholesterolemic Subjects. Am. J. Clin. Nutr..

[51] Larsson S.C., Kumlin M., Ingelman-Sundberg M., Wolk A. (2004). Dietary Long-Chain *n*-3 Fatty Acids for the Prevention of Cancer: A Review of Potential Mechanisms. Am. J. Clin. Nutr..

[52] Sun D., Krishnan A., Zaman K., Lawrence R., Bhattacharya A., Fernandes G. (2003). Dietary N-3 Fatty Acids Decrease Osteoclastogenesis and Loss of Bone Mass in Ovariectomized Mice. J. Bone Miner. Res..

[53] Akasaka H., Ruan K.-H. (2016). Identification of the Two-Phase Mechanism of Arachidonic Acid Regulating Inflammatory Prostaglandin E2 Biosynthesis by Targeting COX-2 and MPGES-1. Arch. Biochem. Biophys..

[54] Coetzee M., Haag M., Claassen N., Kruger M.C. (2005). Stimulation of Prostaglandin E2 (PGE2) Production by Arachidonic Acid, Oestrogen and Parathyroid Hormone in MG-63 and MC3T3-E1 Osteoblast-like Cells. Prostaglandins Leukot. Essent. Fat. Acids.

[55] Kanematsu M., Sato T., Takai H., Watanabe K., Ikeda K., Yamada Y. (2000). Prostaglandin E2 Induces Expression of Receptor Activator of Nuclear Factor-Kappa B Ligand/Osteoprotegrin Ligand on Pre-B Cells: Implications for Accelerated Osteoclastogenesis in Estrogen Deficiency. J. Bone Miner. Res..

[56] Suda K., Udagawa N., Sato N., Takami M., Itoh K., Woo J.-T., Takahashi N., Nagai K. (2004). Suppression of Osteoprotegerin Expression by Prostaglandin E2 Is Crucially Involved in Lipopolysaccharide-Induced Osteoclast Formation. J. Immunol..

[57] Wennberg M., Tornevi A., Johansson I., Hörnell A., Norberg M., Bergdahl I.A. (2012). Diet and Lifestyle Factors Associated with Fish Consumption in Men and Women: A Study of Whether Gender Differences Can Result in Gender-Specific Confounding. Nutr. J..

[58] Galli C., Trzeciak H.I., Paoletti R. (1971). Effects of Dietary Fatty Acids on the Fatty Acid Composition of Brain Ethanolamine Phosphoglyceride: Reciprocal Replacement of *n*-6 and *n*-3 Polyunsaturated Fatty Acids. Biochim. Biophys. Acta Lipids Lipid Metab..

