# Supplementary material for: Plasma Fatty Acids and Quantitative Ultrasound, DXA and pQCT Derived Parameters in Postmenopausal Spanish Women
Source: Nutrients. 2021 Apr 25;13(5):1454. doi: 10.3390/nu13051454 (PMC8146540; doi:10.3390/nu13051454)
Supplement: Supplementary file 1 [file nutrients-13-01454-s001.zip › nutrients-1156629-SI.pdf]

Supplementary Table S1. Bivariate and partial correlations between plasma fatty acids profile and bone mineral density at either the lumbar spine or the hips.

		BMD L2-L4 (g/cm2)	BMD L2 (g/cm2)	BMD L3 (g/cm2)	BMD L4 (g/cm2)	BMD Femoral trochanter (g/cm2)	BMD Femoral neck (g/cm2)
C8:0 Caprylic acid	Spearman Rho	-0.061	-0.057	-0.075	-0.051	-0.054	0.005
	p-Value	0.294	0.32	0.193	0.38	0.351	0.929
C10:0 Decanoic acid	Spearman Rho	-0.119	-0.115	-0.105	-0.113	-0.103	-0.154
	p-Value	0.039	0.046	0.068	0.05	0.074	0.007
	Adjusted coefficient *	-0.094	-0.097		-0.084		-0.115
	p-Value	0.123	0.111		0.171		0.060
C12:0 Lauric acid	Spearman Rho	-0.094	-0.077	-0.093	-0.1	-0.078	-0.125
	p-Value	0.102	0.185	0.108	0.084	0.179	0.031
	Adjusted coefficient *						-0.144
	p-Value						0.018
C14:0 Myristic acid	Spearman Rho	0.018	-0.008	0.02	0.035	0.008	0.052
	p-Value	0.753	0.89	0.732	0.545	0.891	0.366
C16:0 Palmitic acid	Spearman Rho	0.051	0.034	0.044	0.062	0.049	0.075
	p-Value	0.382	0.555	0.448	0.286	0.399	0.194
C18:0 Stearic acid	Spearman Rho	0.039	0.026	0.036	0.037	0.033	0.047
	p-Value	0.503	0.657	0.531	0.528	0.564	0.413
C20:0 Arachidic acid	Spearman Rho	-0.039	-0.037	-0.042	-0.043	-0.021	0.042
	p-Value	0.501	0.527	0.469	0.461	0.718	0.469
C22:0 Docosanoic acid	Spearman Rho	0.05	0.057	0.055	0.028	-0.002	-0.001
	p-Value	0.387	0.326	0.343	0.626	0.978	0.98
C24:0 Lignoceric acid	Spearman Rho	0.004	0.025	-0.008	-0.001	-0.048	-0.075
	p-Value	0.945	0.663	0.884	0.984	0.403	0.194
C16:1 Palmitoleic acid	Spearman Rho	-0.061	-0.064	-0.052	-0.049	-0.044	-0.029
	p-Value	0.292	0.269	0.365	0.399	0.449	0.619
C18:1 cis (n9) Oleic acid	Spearman Rho	-0.104	-0.111	-0.097	-0.08	-0.099	-0.092
	p-Value	0.071	0.055	0.093	0.165	0.087	0.113
C22:1 (n9) Erucic acid	Spearman Rho	-0.021	-0.025	-0.023	-0.017	-0.043	-0.122
	p-Value	0.711	0.661	0.685	0.766	0.46	0.035
	Adjusted coefficient *						-0.114

	p-Value						0.063
C18:3 (n3) Linolenic acid (ALA)	Spearman Rho	0.119	0.109	0.106	0.119	0.026	0.018
	p-Value	0.039	0.058	0.066	0.039	0.654	0.75
	Adjusted coefficient *	0.111			0.123		
	p-Value	0.069			0.044		
C20:5 (n3) Eicosapentenoic acid (EPA)	Spearman Rho	-0.036	-0.046	-0.042	-0.015	-0.009	0.008
	p-Value	0.534	0.428	0.468	0.79	0.875	0.892
C22:6 (n3) Docosahexenoic acid (DHA)	Spearman Rho	0.133	0.128	0.114	0.147	0.083	0.152
	p-Value	0.021	0.026	0.049	0.011	0.151	0.008
	Adjusted coefficient *	0.135	0.148	0.122	0.136		0.132
	p-Value	0.026	0.015	0.045	0.026		0.031
C18:2 (n6) Linoleic acid (LA)	Spearman Rho	-0.014	0.017	-0.018	-0.029	0.014	-0.046
	p-Value	0.805	0.773	0.76	0.613	0.809	0.424
C20:4 (n6) Arachidonic acid (AA)	Spearman Rho	0.054	0.064	0.088	0.015	0.031	0.018
	p-Value	0.355	0.271	0.13	0.802	0.595	0.755

* Partial non parametric correlations adjusted by, age, years since menopause, BMI and vitamin D, calcium and energy intakes.

Supplementary Table S2. Logistic regression of fatty acids associated with low BMD (T≤ - 1 score).

	**Univariate**			**Multivariate**		
	**OR**	**95% CI**	***p*-Value**	**OR**	**95% CI**	***p*-Value**
C8:0 Caprylic acid	2.750	0.023-322.722	0.667			
C10:0 Decanoic acid	2.958	1.131-7.736	0.027	2.412	0.881-6.598	0.087
C12:0 Lauric acid	1.542	0.826-2.878	0.174			
C14:0 Myristic acid	0.851	0.296-2.448	0.764			
C16:0 Palmitic acid	0.925	0.844-1.013	0.093			
C18:0 Stearic acid	0.978	0.922-1.038	0.465			
C20:0 Arachidic acid	1.166	0.894-1.522	0.257			
C22:0 Docosanoic acid	0.581	0.104-3.243	0.536			
C24:0 Lignoceric acid	1.400	0.226-8.677	0.718			
C16:1 Palmitoleic acid	1.100	0.559-2.165	0.782			
C18:1 cis (n9) Oleic acid	1.083	0.996-1.177	0.061			
C22:1 (n9) Erucic acid	0.054	0.000-1823.809	0.584			
C18:3 (n3) Linolenic acid (ALA)	0.742	0.524-1.050	0.092			
C20:5 (n3) Eicosapentenoic acid (EPA)	1.612	0.623-4.169	0.325			
C22:6 (n3) Docosahexenoic acid (DHA)	0.757	0.561-1.023	0.070			
C18:2 (n6) Linoleic acid (LA)	1.044	0.970-1.125	0.250			
C20:4 (n6) Arachidonic acid (AA)	0.902	0.744-1.050	0.184			

OR: Odds ratio; CI: Confidence interval

Multivariate model adjusted by: Age (years), Years since menopause (years) BMI (kg/m^2), Vitamin D (mcrg/day), Calcium intake (mg/day), Energy (kcal/day)
